# A Carapace-Like Bony ‘Body Tube’ in an Early Triassic Marine Reptile and the Onset of Marine Tetrapod Predation

**DOI:** 10.1371/journal.pone.0094396

**Published:** 2014-04-09

**Authors:** Xiao-hong Chen, Ryosuke Motani, Long Cheng, Da-yong Jiang, Olivier Rieppel

**Affiliations:** 1 Wuhan Center of China Geological Survey, Wuhan, Hubei, P. R. China; 2 Department of Earth and Planetary Sciences, University of California Davis, Davis, California, United States of America; 3 Laboratory of Orogenic Belt and Crustal Evolution, Ministry of Education, Department of Geology and Geological Museum, Peking University, Beijing, P.R. China; 4 Center of Integrative Research, The Field Museum, Chicago, Illinois, United States of America; University of Pennsylvania, United States of America

## Abstract

*Parahupehsuchus longus* is a new species of marine reptile from the Lower Triassic of Yuan’an County, Hubei Province, China. It is unique among vertebrates for having a body wall that is completely surrounded by a bony tube, about 50 cm long and 6.5 cm deep, comprising overlapping ribs and gastralia. This tube and bony ossicles on the back are best interpreted as anti-predatory features, suggesting that there was predation pressure upon marine tetrapods in the Early Triassic. There is at least one sauropterygian that is sufficiently large to feed on *Parahupehsuchus* in the Nanzhang-Yuan’an fauna, together with six more species of potential prey marine reptiles with various degrees of body protection. Modern predators of marine tetrapods belong to the highest trophic levels in the marine ecosystem but such predators did not always exist through geologic time. The indication of marine-tetrapod feeding in the Nanzhang-Yuan’an fauna suggests that such a trophic level emerged for the first time in the Early Triassic. The recovery from the end-Permian extinction probably proceeded faster than traditionally thought for marine predators. *Parahupehsuchus* has superficially turtle-like features, namely expanded ribs without intercostal space, very short transverse processes, and a dorsal outgrowth from the neural spine. However, these features are structurally different from their turtle counterparts. Phylogeny suggests that they are convergent with the condition in turtles, which has a fundamentally different body plan that involves the folding of the body wall. Expanded ribs without intercostal space evolved at least twice and probably even more among reptiles.

## Introduction

The modern marine ecological web entails complex interactions among species of multiple trophic levels, from primary producers to apex predators. The relative trophic level of each individual is often measured by a nitrogen isotope fractionation value, δ^15^N [1]. The heavier-than-normal isotope accumulates in the body of predators through predation, thus reaching the highest values in the apex predators. The value cannot be compared across a geographic range because the base concentration of ^15^N depends on the local environment.

It has been observed in the modern marine ecosystem that those predators that feed on marine tetrapods reach higher trophic levels than fish or cephalopod feeders. For example, individuals feeding on tetrapods tend to have higher δ^15^N values than fish or squid eaters in both killer whales [2] and great white sharks [3]. This suggest that marine tetrapods as prey are an essential element that supports the highest trophic level in the modern ocean. Then, it is evident that such a high trophic level did not always exist throughout the history of life because marine tetrapods have a limited stratigraphic range. This raises a question of when in geologic time marine tetrapods as prey species became available, and apex marine predators to feed on them evolved.

Ribs are an essential structure that is common to all vertebrates. They display different morphologies depending on taxonomy, ontogeny, and position along the body axis. Unlike in cervical or sacral ribs, the main bodies of dorsal ribs are largely uniform across taxa, being curved rods with spaces, bridged by intercostal muscles [4], [5]. At least some intercostal space persists even in reptiles with expanded dorsal ribs or body armors, such as *Sinosaurosphargis* [6], *Largocephalosaurus* [7], [8], cyamodontid placodonts [9], ankylosaurs [10], and *Eunotosaurus* [11], although the spaces may be partly closed.

A notable exception is the turtle, whose costal plate grows from the rib and completely eliminates the intercostal space except in *Dermochelys* and *Odontochelys* [6]. We report here a different lineage of marine reptile that independently eliminated the dorsal intercostal spaces through rib expansion, forming a bony ‘body tube’ rather than a carapace.

**Figure 1:**
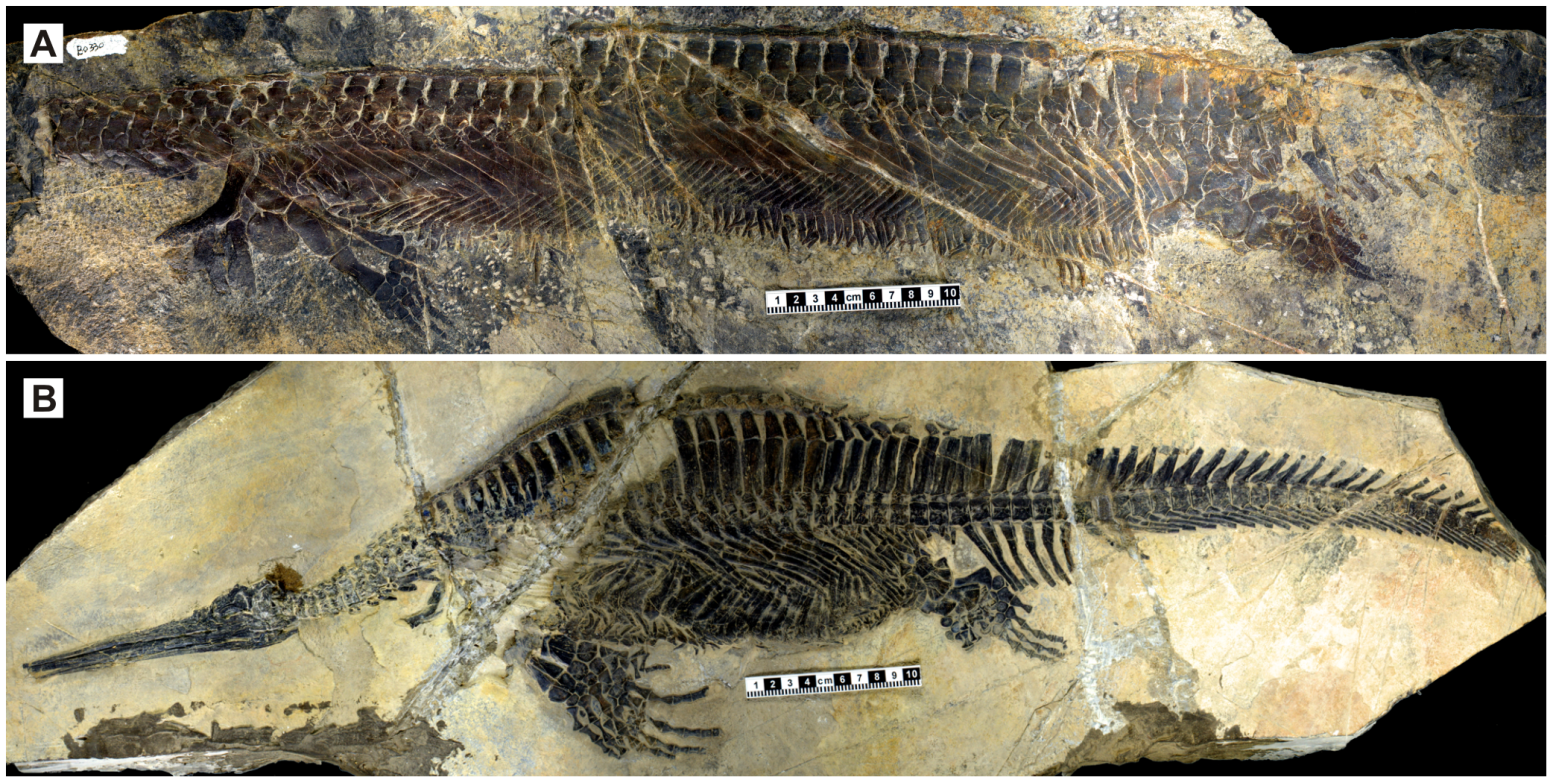
Holotype of *Parahupehsuchus longus* (WGSC 26005) and a specimen of *Hupehsuchus nanchangensis* (WGSC 26004). (A), whole view of WGSC 26005. (B), whole view of WGSC 26004. Scales are 10 cm long.

Hupehsuchia [12] is an enigmatic group of marine reptiles that is endemic to the Lower Triassic of Hubei Province, China (ca. 248 million years ago [13], [14]). Two monotypic genera are known, namely *Nanchangosaurus* [15] and *Hupehsuchus* [16]. A third genus was suggested in the literature but has not been formally named [12]. The group is known for a suite of unusual features, such as an edentulous and beak-like snout, double-layered neural spines, a heavily ossified skeletal construction, and polydactyly [12], [17]. The bizarre body plan of *Hupehsuchus* (Fig. 1B) has led to a controversy about its paleoecology [12], [18].

In 2011, Wuhan Centre of China Geological Survey (WGSC hereafter) undertook a field excavation in Yuan’an County, Hubei Province, China to find Early Triassic marine reptiles. The fieldwork resulted in more than ten specimens of marine reptiles, one of which is reported here as a key species to indicate the onset of marine tetrapod predation, as well as a new example of species bearing turtle-like expansion of ribs.

## Materials and Methods

### Specimens

The specimens observed for the present study are IVPP (Institute of Vertebrate Paleontology and Paleoanthropology, Beijing, China) V3232 (holotype of *Hupehsuchus nanchangensis*) and V4070, WGSC 26004, 26005, and 0940. IVPP V4070 is the specimen that [12] recognized as representing the third genus of Hupehsuchia without formally naming it because of the poor preservation. The WGSC specimens were excavated with proper permit from the Bureau of Land and Resources, China, and are accessioned in the fossil collection at the central facility of WGSC in Wuhan, Hubei Province, China.

### Phylogeny

Phylogenetic analysis of Hupehsuchia has never been conducted before because only two named species had been known. We therefore built a new data matrix containing 25 discrete morphological characters for four ingroup and two outgroup taxa. See Text S1 (Supporting Information) for the matrix and character descriptions. The small matrix size allowed branch and bound searches that are guaranteed to find all most parsimonious trees. We used the computer software PAUP*4b10 and TNT 1.1 for tree searches. Bremer support and bootstrap values (n = 1000) were estimated using TNT 1.1.

### Nomenclatural Acts

The electronic edition of this article conforms to the requirements of the amended International Code of Zoological Nomenclature, and hence the new names contained herein are available under that Code from the electronic edition of this article. This published work and the nomenclatural acts it contains have been registered in ZooBank, the online registration system for the ICZN. The ZooBank LSIDs (Life Science Identifiers) can be resolved and the associated information viewed through any standard web browser by appending the LSID to the prefix “http://zoobank.org/”. The LSID for this publication is: urn:lsid:zoobank.org:pub:0F2EED52-F0A2-4125-B96A-8E39E9854DBE. The electronic edition of this work was published in a journal with an ISSN, and has been archived and is available from the following digital repositories: PubMed Central and LOCKSS.

## Results

### Phylogenetic Analysis

**Figure 2:**
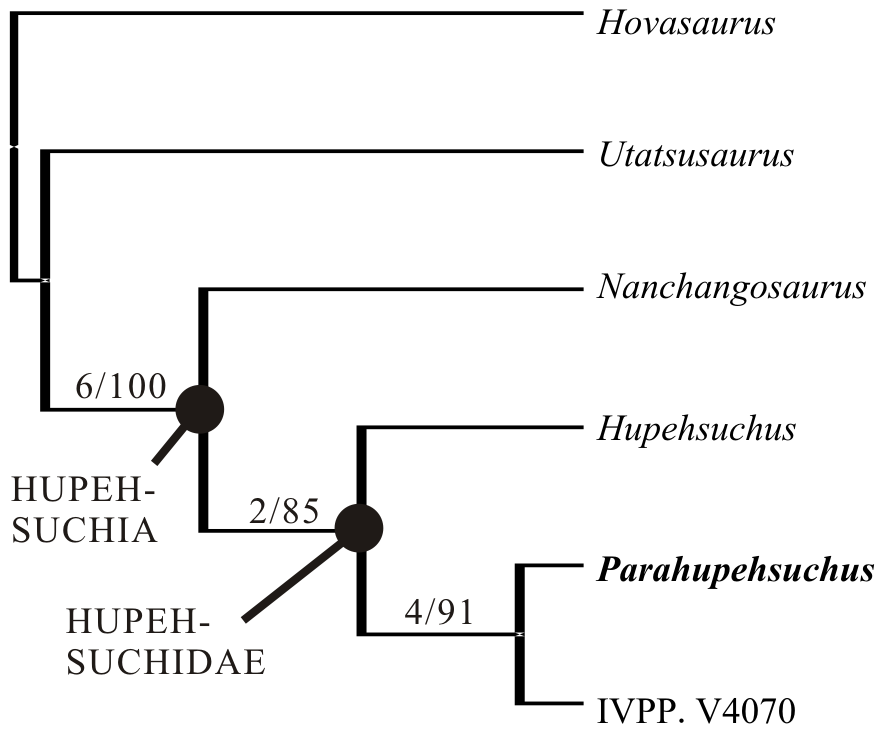
A phylogenetic hypothesis of hupehsuchian relationships. The tree is the single most parsimonious tree (TL = 28, CI = 0.964, RI = 0.957), given the small data matrix. Numbers are Bremer support/bootstrap (n = 1000) values. *Parahupehsuchus* is derived within a well-defined Hupehsuchia. See Text S1 for the data matrix.

PAUP*4b10 and TNT 1.1 both found a single most parsimonious tree (Fig. 2), which is unsurprising given the small number of taxa contained in the data matrix. The tree has TL of 28, CI of 0.964, and RI of 0.957. Bremer support for the basal node of Hupehsuchia is 6, indicating that a large number of unique anatomical features are shared by its members. *Parahupehsuchus* forms a clade with IVPP V4070, from which it differs in many morphological characters as described below. This clade also has a robust Bremer support, with a value of 4.

The data matrix suggests that IVPP V4070 is diagnostic at least to the species level. However, we refrain from naming it, following the wisdom of [12]–the specimen is largely composed of natural molds of bone elements that are not always well defined. We will name the species in the future when describing an additional specimen that is probably conspecific with IVPP V4070.

### Systematic Paleontology

**Systematic hierarchy.**

Reptilia Laurenti, 1768 [19].

Diapsida Osborn, 1903 [20].

Hupehsuchia Carroll and Dong, 1991 [12].

#### Revised diagnosis

Snout elongated, flat, and edentulous; humerus with anterior flange; radiale larger than other proximal carpals; presacral vertebral count exceeding 36; first segments of posterior dorsal neural spines without interspinal space; posterior flange of rib present at least proximally; lateral gastralia boomerang-shaped, pointing anteriorly, with short side directed medially; anterior flange of lateral gastralia overlapping adjacent gastralia.

Hupehsuchidae Young, 1972.

#### Revised Definition

The last common ancestor of *Hupehsuchus* and *Parahupehsuchus*, and all of its descendants.

#### Revised Diagnosis

Second neural spine segment in anterior dorsal region; third layer of dermal armor in dorsal region.

#### Type genus

*Hupehsuchus* Young, 1972.

*Parahupehsuchus longus* gen. et sp. nov.

urn:lsid:zoobank.org:act:0B2F1D4D-0435-496F-B1C0-6913F265240F.

#### Etymology

Generic name is a combination of παρά (Gr. near), hupeh (alternate spelling for Hubei), and ΣοU˜χος (Gr. name for the Egyptian crocodile deity Sobek). Specific name is from λονγοσ (Gr. long).

#### Holotype

**Figure 3:**
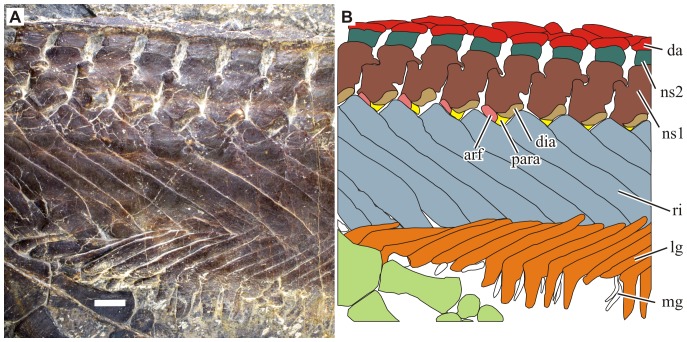
Anterior dorsal region of *Parahupehsuchus longus* (WGSC 26005). Bone identifications: arf, anterior rib facet extending from the parapophysis; da, dermal armor; dia, diapophysis of the neural arch; f, forelimb; lg, lateral gastralia; mg, median gastralia; ns1, first segment of neural spine; ns2, second segment of neural spine; para, the main facet of parapophysis; ri, rib. Scale is 1 cm. Note that ribs and gastralia overlap in a complex manner and the double rib articulation prevents rib motion.

**Figure 4:**
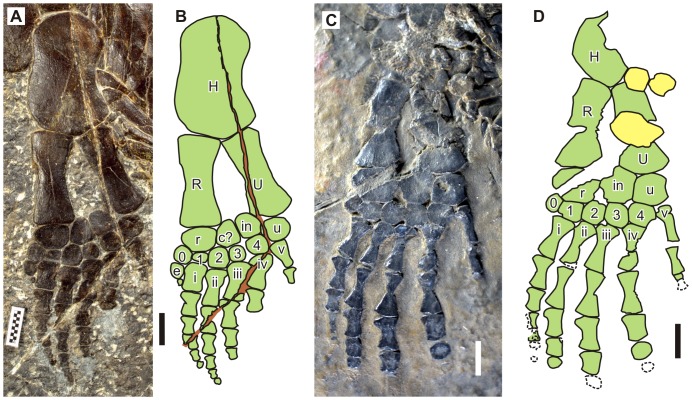
Forelimbs of the holotypes of *Parahupehsuchus longus* (WGSC 26005) and *Hupehsuchus nanchangensis* (IVPP V3232). (A), left forelimb of WGSC 26005. (B), map of A. (C), left forelimb of IVPP V3232. (D), map of C. Bone identifications: c?, bone identified as centralia by [12]; e, extra anterior metacarpal; H, humerus; in, intermedium; R, radius; r, radiale; U, ulna; u, ulnare; 0–4, distal carpals; i–v, metacarpals. Scales are 1 cm.

**Figure 5:**
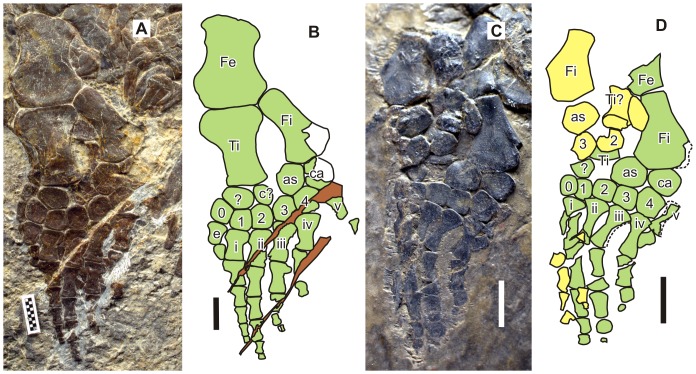
Hind limbs of the holotypes of *Parahupehsuchus longus* (WGSC 26005) and *Hupehsuchus nanchangensis* (IVPP V3232). (A), left hind limb of WGSC 26005. (B), map of A. (C), left hind limb of IVPP V3232. (D), map of C. Bone identifications: as, astragalus; c?, bone identified as centralia by [12]; ca, calcaneum; e, extra anterior metatarsal; Fe, femur; Fi, fibula; Ti, tibia; 0–4, distal tarsals; i–v, metatarsals, ? suspected neomorph. Scales are 1 cm.

WGSC 26005 (Figs. 1A, 3, 4A–B, 5A–B).

#### Diagnosis

Dorsal rib with extensive anterior and posterior flanges; dorsal intercostal space absent except near girdles; second rib facet on neural arch for anterior rib; trunk long, with about 38 dorsal vertebrae; ribcage with more or less unchanged dorsoventral depth; proximal carpal/tarsal row with extra element; extra anterior element in each of distal carpal, metacarpal, distal tarsal, and metatarsal rows.

#### Locality and Horizon

From the upper Spathian (Lower Triassic) Jialingjiang Formation, exposed in Yuan’an County, Hubei Province, China [14].

### Description

#### General design

The preserved length of the skeleton is about 73 cm, of which the trunk makes up about 50 cm. The body is slender, being longer but narrower than in *Hupehsuchus* (Fig. 1). The depth of ribcage is about 65 mm throughout the trunk, giving rise to a ‘parallel-sided’ ribcage unlike the swollen one in *Hupehsuchus*. The difference in the degree of body elongation is reflected in vertebral count: there are 38 dorsal vertebrae in *Parahupehsuchus*, as opposed to about 28 in *Hupehsuchus* [12] and IVPP V4070. Five cervical, two sacral, and 11 caudal vertebrae are preserved but the cervical and caudal counts are incomplete, preventing comparisons with other hupehsuchians.

#### Rib

The dorsal ribs are the most peculiar of all bones in the specimen. It has anterior and posterior flanges that span the entire exposed length (Fig. 3). The extensive posterior flange overlies the posterior adjacent rib. The posterior flange also exists in some ribs of *Hupehsuchus* but only proximally. Given that only the external surface is exposed, it is unknown at this point if the medial side of the rib was also flat as the exterior, or whether it was T-shaped in cross-section as in turtles [6] and *Eunotosaurus* [11].

Each dorsal rib articulates with two adjacent vertebrae with a unique configuration (Fig. 3B). The most proximal few centimeters of the ribs are thick and lack both the anterior and posterior flanges. There is a single rib head, which is much broader than the corresponding diapophysis on the neural arch and bears two facets, one proximally and the other posteriorly. The posterior surface is only recognizable in a limited number of ribs because it is ventrally inclined and not obvious in dorsal view. The wide proximal rib facet articulates with the diapophysis (Fig. 3B, dia) that is narrower than itself but its anterior end seems to connect to the parapophysis on the centrum (Fig. 3, para and arf), which has an unusual shape; it has two articular facets, of which the postero-ventral one (Fig. 3B, para) seems to be homologous with the reptilian parapophyses and is almost confluent with the diapophysis–thus, this part of the parapophysis and diapophysis together form a synapophysis. There is an additional facet that stretches antero-dorso-medially from the main facet, forming a band of rough surface (Fig. 3B, arf). This additional facet articulates with the posterior facet of the rib that lies anteriorly. We will refer to this additional facet as the anterior rib facet hereafter. The anterior rib facet is elevated dorsally above the average dorsal margin of the centrum. Whereas the overlapping of ribs alone may have permitted some degree of sliding between ribs, this double articulation must have limited any mobility of these ribs. For the same reason, the longitudinal orientation of the ribs could not have been significantly different from what is preserved, at least proximally; the ribs are preserved perpendicular to the diapophyses, and parallel to the anterior rib facet. Such a double articulation is not known in *Hupehsuchus*. IVPP V4070 is too poorly preserved for the examination of the feature.

The synapophysis and anterior rib facet together form a shallow V-shaped articular surfaces for ribs in both dorsal and lateral views. The V is not tilted in lateral view because of the raised position of the anterior rib facet. Thanks to this configuration, the proximal parts of the ribs were not rotated around their respective axes, i.e., the parasagittal section of the rib flanges was nearly horizontal without pitching; note that this is not a cross-section perpendicular to the rib axis. This allowed the ribs to form a smooth tube in combination.

Another important implication of the rib morphology is that there was no space for intercostal muscles, which must have been largely absent. Such an absence may explain the reason why the dermal ossicles are not found above the ribs when the animal clearly had a mechanism to form such ossifications. It is possible that thick dermis covered the ribs but there is no anatomical feature preserved to either reject or support such an hypothesis.

#### Gastralia

The ribs are extensively overlapped by the gastralia distally but it is unclear if the ribs and gastralia articulated with each other. If such an articulation is absent, then the degree of overlap may have been exaggerated through flattening of the body trunk during fossilization. The overlap between the lateral gastral elements and the distal parts of ribs is commonly seen in hupehsuchian specimens that are exposed in lateral view, i.e., those specimens that experienced compaction in bilateral direction during fossilization. However, the lateral gastral elements always lie external to the ribs. Such consistency in preservation posture across specimens is not expected unless at least the distal tip of the lateral gastral elements lay externally to the ribs in life, forming a bony tube.

There are three parts to the gastralia, namely a pair of lateral gastral elements that are flat and boomerang-shaped, and a single median gastral element that is much smaller and V-shaped. The median gastral element is round in cross-section, unlike its lateral counterpart. The bend of the boomerang of a lateral element is positioned anteriorly whereas the valley of v in a median element is pointing posteriorly. When articulated, the three together form a loose Σ shape in ventral view. *Hupehsuchus* also has a similar condition, with a pair of large lateral and a small median gastral elements. The lateral element was interpreted as the median one by [12].

Different rows of gastralia overlap with each other extensively, with the posterior element positioned externally to the anterior. The way they overlap is in the opposite direction to the pattern of rib overlap, where the posterior element is internal to the anterior one. This counter-overlapping pattern between ribs and gastralia must have further limited the flexibility of the trunk, even if the overlap between ribs and gastralia was less than what is preserved.

#### Neural spine

Despite the slender trunk, the dorsal neural spines of *Parahupehsuchus* are bipartite (Figs. 1A, 3), with a second segment above the original neural spine as in *Hupehsuchus* [12](Fig. 1B). The second segment is continuous with the first layer of dermal ossicles without a clear suture. Compared to the first segment, the height of the second segment is low, being less than half of the former. Also, the second segment is slightly narrower than the first segment–in *Hupehsuchus*, the base of the second segment is narrower than the top of the first segment only posteriorly in the trunk.

The second segment is already present in the most anterior cervical vertebra in the specimen. It is also present at least in the first six caudal vertebrae–the relevant parts are poorly preserved in more posterior vertebrae. In other words, every well-preserved neural spine in the specimen has a second segment. The second segment of *Hupehsuchus* is limited mostly to the dorsal region [12].

#### Dermal ossicles

There are up to three layers of dermal ossicles in the trunk (Fig. 3). The first layer extends immediately above the neural spine, occupying the entire width of the latter. These ossicles are somewhat triangular, pointing upward. The space between the first-layer ossicles is occupied by the second-layer ossicles, which are smaller and point downward to fit into the triangular space between the first layer elements. The third layer ossicles lie above the first two layers. Each third-layer ossicle is larger than the ones below, and usually spans two to three vertebral segments.

As with the second segment of neural spine, dermal ossicles are present throughout the specimen when the relevant part is preserved. Thus, even the most anterior cervical vertebra is associated with the first layer of dermal ossicle, and so are at least the first six caudal vertebrae. The second layer elements are also present as long as there is a gap to fill between a pair of first layer elements. The third layer, however, has a more restricted distribution. The most cranial third layer element is above the eighth to tenth dorsal vertebrae, whereas the most caudad one is above the last dorsal and the two sacral vertebrae. This distribution pattern is very different from the more limited range in *Hupehsuchus*–dermal ossicles are present only between the last cervical and second caudal vertebrae, and the third layer is present between the 13th and last dorsal vertebrae in the genus.

#### Forelimb

The forelimb is flipper-shaped (Fig. 4A–B), unlike the paddle-shaped forelimb of IVPP V4070 or the polydactylous specimen of [17]. The phalangeal width clearly becomes narrower toward the tip of the manus. This is in contrast with the condition in *Hupehsuchus*, where the width reduction is almost absent (Fig. 4C–D). The manus is slightly longer than the zeugopodium. This again differs from *Hupehsuchus* whose manus is almost twice as long as the zeugopodium.

There is an additional anterior digit (digit ‘0′) with a distal carpal, metacarpal and very small first phalanx. The first phalanx of manual digit 0 is so far unknown in *Hupehsuchus*. The preserved digital formula including this digit is (1)-5-5-3-1-1 but it is likely that distal phalanges are missing from digits 3 to 5 because the most distal elements are still large compared to those of digits 1 and 2. Further preparation of the relevant parts of the fossil using carbide needles, however, did not reveal any additional element. The type specimen of *Hupehsuchus nanchangensis* has a phalangeal formula of (0)-4-4-4-4-2, so the longest digits are longer in *Parahupehsuchus*. The manus of the polydactylous specimen as figured by [17] seems to preserve a phalangeal formula of at least (3)-(4)-5-4-4-5-4, which is clearly different from the present formula in having another additional digit and increased numbers of phalanges posteriorly. The manual phalangeal formula of IVPP V4070 cannot be established with confidence because its forelimbs are incomplete distally.

The arrangement of the proximal carpals is puzzling. There is an extra element between the radiale and intermedium. A similar element in IVPP V4070 was identified as a centrale by [12] and lateral centrale by [17]. However, given that each of proximal and distal carpal rows has an extra element, it is also possible that this proximal element is a neomorph that was derived anteriorly from the intermedium. Also, *Parahupehsuchus* is more derived than *Hupehsuchus* (Fig. 2), which clearly lacks the suspected centrale. See below for further discussion in the section of hind limb.

Manual digits 1 and 2 of *Parahupehsuchus* are more tightly ‘bundled’ than the rest of the digits and converge distally. A similar bundling is seen in the type specimen of *Hupehsuchus nanchangensis* (Fig. 4C), so the condition is probably natural and not an artifact of preservation, unlike the interpretation presented in figures 5 and 11 of [12]. Such bundling is not obvious in IVPP V4070 or the polydactylous specimen described by [17].

#### Hind limb

The hind limb of *Parahupehsuchus* closely resembles its forelimb–it is flipper-shaped and its phalangeal width decreases rapidly toward the tip (Fig. 5A–B). Also, there is digit 0 with a distal tarsal, metatarsal, and the first phalanx. This phalanx, however, is larger than in the forelimb. The phalangeal formula is (1)-5-5-4-2-1 but distal phalanges are likely missing from digits 4 and 5. This is similar to the formula for IVPP V4070, which is (0)-4-5-5-?-1 but this latter hind limb is fan-shaped unlike the flipper-shaped hind limb of *Parahupehsuchus*. The phalangeal formula for the pes of *Hupehsuchus nanchangensis* is obscure; the distal end of the hind limb of the holotype (Fig. 5C, D) appears to be incompletely prepared. The preserved phalangeal formula in the polydactylous specimen of [17] is (4)-5-6-6-4-3, which is unique among hupehsuchians in having more than five phalanges in the longest digits (hyperphalangy).

The tibia is wider proximally than distally, as in most marine reptiles. The tibia of *Hupehsuchus* was previously reconstructed to be similar to the fibula [12], but an alternative interpretation may be that the element that was interpreted as the tibia is a laterally-flipped fibula, as in Fig. 5D. Notably, the hind limb of *Parahupehsuchus* is only slightly shorter than the forelimb–in *Hupehsuchus*, the forelimb is much larger than the hind limb (Figs. 1, 4 and 5).

Two additional proximal tarsals exist, rather than one as in the forelimb. Both are located distal to the tibia, with the posterior bone being smaller than the anterior element. IVPP V4070 also has two additional proximal tarsals but their relative size is the opposite of the condition in *Parahupehsuchus* because the posterior element is larger in that specimen [12]. The homology of these two bones is again debatable. The posterior element may be a centrale as suggested by [12] and [17]but that would still leave the anterior element as a neomorph, which is somehow more prominent than the suspected centrale in *Parahupehsuchus*. Given that the condition cannot be explained by involving at least one neomorph, the simplest interpretation may be to identify both of them as neomorphs. This, together with the appearance of the suspected centrale only in the derived member of Hupehsuchia, suggests that the bone may indeed be a neomorph. If so, the extra proximal carpal may also be a neomorph.

## Discussion

The body tube of *Parahupehsuchus* provided the trunk with very limited flexibility despite its slender appearance. It undoubtedly restricted possible methods of locomotion. The limbs of *Parahupehsuchus* are too small relative to the body to be the main propulsive organs. The tail of *Parahupehsuchus* is unknown but, given that hupehsuchians generally have tails that are longer than the rest of the body, it is likely that *Parahupehsuchus* also relied on its tail for propulsion. Then, the swimming style of this genus likely resembled that of extant crocodylians, which have a stiff trunk and use the long tail for aquatic propulsion. The steering method, however, may have been different. The flipper shape of the limbs indicate their use as steering device as in many cetaceans, rather than drag-inducing maneuvering device as in the paddles of crocodiles and some aquatic turtles.

Another limitation imposed by the stiff trunk concerns the mechanics of respiration. The dorsal rib of *Parahupehsuchus* cannot rotate or move fore-and-aft because of the skeletal structures. Furthermore, there is no space for intercostal muscles that would move the rib. Therefore, it is impossible to change the volume of the body cavity to produce pressure differentiation for respiration through rib motion, unlike in many tetrapods [21]. Two other mechanisms used by *Crocodylus* to induce pressure differentiation in the chest is the translation of gastralia and pelvic rotation using abdominal muscles, and visceral movement by the diaphragmaticus muscle [22]. Of the two, the gastralia translation also seems impossible in *Parahupehsuchus* given the large overlap between the gastralia and ribs. This leaves the use of diaphragmaticus muscle and visceral movement, also known as hepatic piston [21], as the only alternative. This mechanism is not as important in *Crocodylus* as previously believed [22] but was a major mechanism among dinosaurs [23]. The holotype and only specimen of *Parahupehsuchus longus* does not preserve any positive or negative evidence regarding this interpretation.

The body tube of *Parahupehsuchus* is relevant to two ongoing debates, namely the speed of biotic recovery after the end-Permian mass extinction and the evolution of widely expanded ribs in reptiles, as in turtles. We will discuss them separately below.

### Tetrapod Predation and Triassic Recovery

As mentioned in the Introduction, the modern trophic structure in the marine ecosystem did not always exist through geologic time, leaving the question of when it originated. Particularly interesting is the appearance of tetrapod eaters, which defines the highest trophic level in the modern marine ecosystem, together with their tetrapod prey. Tetrapods did not appear until the Carboniferous, and the only truly marine tetrapod in the Paleozoic were the mesosaurids of the Early Permian. This group was endemic to the Irati and White Hill Seas that were enclosed [24], [25], and never invaded the open ocean. The appearance of open-ocean reptiles had to wait until the Triassic [26], in the new ecosystem that evolved after the devastating end-Permian mass extinction. The Nanzhang-Yuan’an fauna is one of the best preserved of such earliest marine tetrapod faunas of the Early Triassic.

The stiffened body trunk of *Parahupehsuchus* most likely had an anti-predatory function. The body tube is not a proper carapace because it does not form an outer shell of the body, exposing epaxial, pectoral, and pelvic muscles outside. However, the tube directly protects the internal organs from predators. Moreover, there were few or no intercostal muscles, so much of the trunk lacked exposed muscles that required protection. Furthermore, *Parahupehsuchus* has at least one row of three-layered dermal ossicles above the neural spines, where the external muscle mass is concentrated, suggesting that the dermal ossicles were protecting most of the exposed muscular mass–this marks a clear contrast with saurosphargids, which have a large mass of epaxial muscles overlying the long transverse processes to protect. Therefore, despite the limited extent of dermal ossicles, the body of *Parahupehsuchus* was well protected. The body plan of hupehsuchians in general is toward building a heavily ossified skeleton that would make ingestion and digestion by predators difficult. We interpret the condition in *Parahupehsuchus* as further development of this anti-predation structure. An alternative interpretation for the body tube may be an anti-pressure device for deep diving. However, it is unlikely that hupehsuchian were deep divers. Pachyostosis and increased bone density are common among marine invaders [27], [28] and is expected to ballast the body against the movement of water, such as wave actions near the coastline. The added bone mass likely provides negative buoyancy even with air in the lungs. The skeleton of deep divers, in contrast, tends to have less bone mass [28], [29] and such histological adaptations as spongy cortex bones [30]. Moreover, a solid body trunk is unnecessary for deep diving tetrapods–they experience various degrees of thoracic collapse during diving [31] except in sea turtles, and their internal organs are adapted to withstand the collapse.

Body protection in hupehsuchians suggests that there was a large predator that lived with these relatively small marine tetrapods. Among the new collection from Yuan’an County is a partial skeleton of a large unidentified eosauropterygian (WGSC 0940). The specimen is estimated to have been about 3–4 meters long. Such a sauropterygian predator would be sufficiently large to bite the trunk of *Parahupehsuchus*. Apart from *Parahupehsuchus*, three additional species of hupehsuchians [12], the ichthyopterygian *Chaohusaurus* [32], and two pachypleurosaurs *Hanosaurus* [33], [34] and *Keichousaurus* [14], [35], are known in the Nanzhang-Yuan’an fauna. All of them are about 1 m or less in total length–note that marine reptiles tend to have long tails so the trunk is much shorter and narrower in these reptiles than in the marine mammals of the same total length. Unlike heavy-built hupehsuchians, *Chaohusaurus* was lightly built and the pachypleurosaurs had moderately heavy skeletons. Then, there were at least seven species of potential prey marine reptiles with various degrees of body protection, together with at least one large predator. Notably, no fish fossil is known in the Nanzhang-Yuan’an fauna despite the abundance of marine reptiles, narrowing the prey choice for the large sauropterygian. Then, it is most likely that there was predation pressure upon these smaller marine reptiles.

The composition of the Nanzhang-Yuan’an fauna suggests that marine tetrapods potentially suitable as prey already existed in the Early Triassic, together with their predator. Then, a marine trophic structure similar to the modern one was already being established in the late Early Triassic, only about four million years after the end-Permian mass extinction. This timing is earlier than previously suggested [36]. Recovery after the end-Permian mass extinction was probably faster for marine predators than previously thought [37] to allow the emergence of such a new trophic level that did not exist before the extinction.

### Evolution of Rib Expansion

The skeleton of *Parahupehsuchus* shares three similarities with the turtle shell: expanded ribs without intercostal spaces, short transverse processes, and dorsal outgrowth of the neural spines. However, these features have different structures than those of turtles– for example the ribs of *Parahupehsuchus* overlap extensively and the neural spine outgrowth is fused with dermal armor, unlike in turtles. Also, hupehsuchians lack the folding of the body wall that limits the ribs to the axial domain of the body trunk in turtles [6], [38], marking a fundamental difference in the body plan. Within Hupehsuchia, extensive rib expansion is known only in *Parahupehsuchus* and IVPP V4070, both of which are derived members (Fig. 2). Moreover, some intercostal spaces still remain in the stem-turtle *Odontochelys* [39]. Therefore, elimination of intercostal space is not homologous between *Parahupehsuchus* and turtles.

It is not impossible that the genetic foundation for rib expansion may be shared between the two without being expressed in intermediate taxa, as has been argued for the body wall folding in *Sinosaurosphargis*, cyamodontid placodonts, and turtles [6]. However, the fundamental difference in body plan due to the lack of body wall folding casts doubt on a close phylogenetic relationships between the two. Histological comparison is unfortunately impossible without damaging the holotype.

The present specimen suggests that rib expansion may not have been as rare among reptiles as previously believed. Overlapping ribs from extreme expansion evolved convergently at least twice and probably more times among reptiles. Most reptile groups with expanded ribs occurred in the marine Triassic of South China, between about 248.5 and 233.5 million years ago [13]. Regardless of whether there is a common genetic mechanism underlying this feature, it was at least expressed separately in each lineage. It is then possible that selection favored the feature because of common environmental factors. Candidates include chemical conditions, such as calcium availability, and biological factors, such as predation pressure. Future studies can test this hypothesis from multiple angles.

## Supporting Information

Text S1 Data matrix for phylogenetic analysis.
(DOCX)
