# Supplementary material for: A Carapace-Like Bony ‘Body Tube’ in an Early Triassic Marine Reptile and the Onset of Marine Tetrapod Predation
Source: PLoS One. 2014 Apr 9;9(4):e94396. doi: 10.1371/journal.pone.0094396 (PMC3981804; doi:10.1371/journal.pone.0094396)
Supplement: Text S1 — Data matrix for phylogenetic analysis. (DOCX) [file pone.0094396.s001.docx]

**Text S1.** Data matrix for phylogenetic analysis of Hupehsuchia.

**Data matrix**

|  | 1 | 2 | 3 | 4 | 5 | 6 | 7 | 8 | 9 | 10 | 11 | 12 | 13 | 14 | 15 | 16 |
| --- | --- | --- | --- | --- | --- | --- | --- | --- | --- | --- | --- | --- | --- | --- | --- | --- |
| Hovasaurus | 0 | 0 | 0 | 0 | 0 | 0 | 0 | 0 | 0 | 0 | 0 | 0 | 0 | 0 | 0 | 0 |
| Utatsusaurus | 0 | 1 | 0 | 0 | 1 | 1 | 0 | 2 | 0 | 0 | 0 | 0 | 0 | 2 | 0 | 0 |
| Nanchangosaurus | 1 | 1 | 1 | ? | 1 | 1 | ? | ? | ? | ? | ? | ? | ? | 2 | 0 | 1 |
| Hupehsuchus | 1 | 1 | 1 | 1 | 1 | 1 | 1 | 2 | 1 | 0&1 | 0 | 1 | 1 | 2 | 1 | 1 |
| Parahupehsuchus | ? | ? | ? | 1 | 1 | 1 | 1 | 1 | 1 | 1 | 1 | 1 | 1 | 2 | 1 | 1 |
| IVPP.V4070 | ? | ? | ? | ? | 1 | 1 | 1 | 1 | 1 | 1 | 1 | 0 | 1 | 2 | 1 | 1 |
|  |  |  |  |  |  |  |  |  |  |  |  |  |  |  |  |  |
| (… continued) | 17 | 18 | 19 | 20 | 21 | 22 | 23 | 24 | 25 |  |  |  |  |  |  |  |
| Hovasaurus | 0 | 0 | 0 | 0 | 0 | 0 | 0 | 0 | 0 |  |  |  |  |  |  |  |
| Utatsusaurus | 0 | 0 | 0 | 0 | 0 | 0 | 0 | 0 | 0 |  |  |  |  |  |  |  |
| Nanchangosaurus | 1 | ? | ? | 1 | ? | ? | 1 | 0 | 0 |  |  |  |  |  |  |  |
| Hupehsuchus | 1 | 0 | 1 | 1 | 1 | 1 | 1 | 1 | 0 |  |  |  |  |  |  |  |
| Parahupehsuchus | 2 | 1 | 1 | 1 | 1 | 1 | 1 | 1 | 1 |  |  |  |  |  |  |  |
| IVPP.V4070 | 2 | 1 | ? | ? | ? | ? | 1 | ? | 1 |  |  |  |  |  |  |  |

**Characters and states**

1. Snout, flattened: (1) false; (2) true

2. Snout, elongated much beyond external naris: (1) false; (2) true

3. Teeth: (1) small; (2) edentulous

4. Clavicle, proximal flattened expansion: (1) absent; (2) present

5. Humerus, anterior flange: (1) absent; (2) present

6. Ulna, distal end wider than or as wide as proximal end: (1) false; (2) true

7. Manual pisiform: (1) present; (2) absent

8. Manual centralia: (1) present; (2) dislocated proximally; (3) absent

9. Radiale larger than other proximal carpals: (1) false; (2) true

10. Extra anterior distal carpal: (1) absent; (2) present

11. Extra anterior metapodial: (1) absent; (2) present

12. Digits 1-3 'bundled': (1) false; (2) true

13. Manual digit 1 hyperphalangeal and with max number of phalanges: (1) false; (2) true

14. First haemal arch at vertebral number: (1) less than 30; (2) about 37; (3) 41 or higher

15. Anterior dorsal neural spine, second segment: (1) absent; (2) present

16. Posterior dorsal neural spine, first segment, interspinal space: (1) present; (2) absent

17. Rib, posterior flange: (1) absent; (2) present proximally; (3) present extensively

18. Ribcage, depth: (1) swollen midway; (2) semi-constant

19. Gastralia, anterior flange overlapping anterior gastralia: (1) absent; (2) present

20. Lateral gastralia boomerang-shaped, pointing anteriorly: (1) absent; (2) present

21. Median gastralia v-shaped, pointing posteriorly: (1) absent; (2) present

22. Extensive overlap between dorsal ribs and lateral gastralia: (1) absent; (2) present

23. Dermal armor above dorsal neural spine, first layers: (1) absent; (2) present

24. Dermal armor above dorsal neural spine, third layer: (1) absent; (2) present

25. Dermal armor above caudal vertebrae with haemal arches: (1) absent; (2) present

**Unambiguous synapomorphies**

Hupehsuchia: 1, 3, 16, 17, 20, and 22.

Hupehsuchidae: 15 and 24.

Parahupehsuchus + IVPP 4070: 8, 11, 17, 18, and 25.
